# Gag-Gag Interactions Are Insufficient to Fully Stabilize and Order the Immature HIV Gag Lattice

**DOI:** 10.3390/v13101946

**Published:** 2021-09-28

**Authors:** Ipsita Saha, Benjamin Preece, Abby Peterson, Haley Durden, Brian MacArthur, Jake Lowe, David Belnap, Michael Vershinin, Saveez Saffarian

**Affiliations:** 1 Laboratory of Cell and Developmental Signaling, Center for Cancer Research, National Cancer Institute, National Institutes of Health, Frederick, MD 21702, USA; ipsita.saha@utah.edu; 2 Center for Cell and Genome Science, University of Utah, Salt Lake City, UT 84112, USA; preece.benjaminj@gmail.com (B.P.); petersonabby99@gmail.com (A.P.); hdurden84@gmail.com (H.D.); briandmacarthur@gmail.com (B.M.); nano.bio.geek@gmail.com (M.V.); 3 Department of Physics and Astronomy, University of Utah, Salt Lake City, UT 84112, USA; 4 School of Biological Sciences, University of Utah, Salt Lake City, UT 84112, USA; jakelowe03@hotmail.com (J.L.); David.Belnap@utah.edu (D.B.); 5 Department of Biochemistry, University of Utah, Salt Lake City, UT 84112, USA

**Keywords:** HIV, immature lattice, lattice dynamics

## Abstract

Immature HIV virions harbor a lattice of Gag molecules with significant ordering in CA-NTD, CA-CTD and SP1 regions. This ordering plays a major role during HIV maturation. To test the condition in which the Gag lattice forms in vivo, we assembled virus like particles (VLPs) by expressing only HIV Gag in mammalian cells. Here we show that these VLPs incorporate a similar number of Gag molecules compared to immature HIV virions. However, within these VLPs, Gag molecules diffuse with a pseudo-diffusion rate of 10 nm^2^/s, this pseudo-diffusion is abrogated in the presence of melittin and is sensitive to mutations within the SP1 region. Using cryotomography, we show that unlike immature HIV virions, in the Gag lattice of VLPs the CA-CTD and SP1 regions are significantly less ordered. Our observations suggest that within immature HIV virions, other viral factors in addition to Gag, contribute to ordering in the CA-CTD and SP1 regions.

## 1. Introduction

Human immunodeficiency virus (HIV) is the causative agent of acquired immunodeficiency syndrome (AIDS). AIDS has claimed upward of 30 million lives over the past 30 years and while the disease can be kept in check using potent antivirals, there remains no cure for AIDS. HIV-1 developing resistance to antivirals is an increasingly significant problem which highlights the need for continued development of new antivirals that exploit novel targets to inhibit HIV-1 replication [1]. HIV-1 virions are released from infected cells as immature virions, mainly characterized by a lattice of HIV Gag proteins anchored to the inner leaflet of the virion membrane. During maturation, this lattice is transformed by successive actions of the 22 kD HIV protease dimer [2,3]. The action of the protease, leads to formation of a fullerene cone structure that encapsulates the genomic RNA creating the mature core within the infectious virion [4,5]. Not surprisingly, most of the antivirals available for treatment result in abnormalities in the formation of the mature core. Specifically, protease inhibitors result in lack of proteolysis trapping the lattice in the immature form [6] and maturation inhibitors result in formation of aberrant cores [1,7,8]. A detailed molecular understanding of the maturation process is a primary focus of significant current research [9,10,11]. It is a common view that the immature HIV Gag lattice is a critical starting point for maturation and therefore interactions which result in stabilization of the immature lattice, including the cellular cofactor IP6 have been of great interest [12,13,14].

Immature HIV virions released from infected cells, package ~2000 copies of Gag [15]. The Gag polyprotein has a series of folded domains which include MA, CA with separate CA-NTD and CA-CTD regions as well as SP1 and NC. To become infectious, HIV protease needs to access and cleave the junctions between MA and CA as well as CA and SP1 regions [2], among other cleavage sites. Binding of maturation inhibitors within the immature lattice suggests that the observed order within the Gag lattice plays a crucial role during the proteolysis of Gag which leads to the formation of HIV mature cores [1].

Tomogram averaging cryotomography has resolved major interactions between Gag molecules within the lattice, which shows each molecule having interactions with at least 5 other Gag molecules. Specifically each Gag molecule is stabilized within the lattice through interactions in its CA-NTD domain which forms the basis of the Gag hexagonal structure [16,17]. Further, Gag interacts through a dimeric interaction at its CA-CTD domain, followed by interactions within the folded SP1 six helix bundle [18,19]. These interactions suggest significant Gag avidity within the immature lattice. In addition to the identified Gag-Gag interactions, it has been shown that IP6, which makes ionic contacts with residues within the Gag hexamer, further stabilizes the immature Gag lattice [12,13,14].

HIV Gag expressed within mammalian cells is sufficient for the release of Gag virus- like particles (VLPs) [20]. These particles have similar sizes and appear identical to immature HIV virions in thin section TEM imaging [21,22]. This points to Gag-Gag interactions having a major role in assembly of the immature lattice. In vitro (absent the cell context), the full length HIV Gag does not assemble into spherical particles with sizes similar to HIV [23], however partial deletion of the MA domain and p6 along with addition of RNA results in assembly of fully spherical structures resembling the immature lattice of HIV as observed in cryotomography [24,25]. Crystal structures obtained from the CA-CTD-SP1 region of Gag were also shown to form an identical ordered structure to the lattice observed in immature HIV virions [18]. Given the extent of all the interactions present within Gag, assembly of Gag at sufficient concentration in the mammalian cells is suggested to release VLPs with similar order as observed in the immature virions [25].

We previously established a method based on iPALM [26] to further characterize HIV Gag VLPs produced by expressing Gag fused to the photoswitchable protein Dendra2 after the p6 domain (Gag-Dendra2). We then found that each Gag-Dendra2 VLP incorporates an average of ~2000 Gag-Dendra2 molecules similar to the number of Gag molecules incorporated within immature HIV virions [15,27]. In addition we found that Gag molecules within these VLPs had detectable dynamics observed both through iPALM and biochemical measurements, although at that time we did not quantify this diffusion [27]. Given the extensive multi layered Gag lattice, which includes hexagonal lattice formation between CA-NTD as well as CA-CTD and the six-helix bundle of SP1, the observed Gag dynamics within the lattice of purified VLPs was perplexing. In this manuscript, we have further developed the methodology to quantify the dynamics within the Gag lattice of purified VLPs and examine the effects of membrane as well as protein-protein interactions on these dynamics. In addition, we present cryotomography data on differences between Gag VLPs and immature HIV virions that can partially explain the observed dynamics.

## 2. Materials and Methods

### 2.1. VLP Harvest

The 293 cells were grown in DMEM media supplemented with 10% FBS at 37 °C. The designated plasmids are transfected using Lipofectamin 2000 (Thermo Fisher Scientific, Waltham, MA, USA). The supernatant is collected 36 h post transfection. The cells in the supernatant were separated through centrifugation (F33V, from Champion, Gwinnett, GA, USA) at 5000 rpm for 15 min. The supernatant was filtered using 0.4 um filters. The VLPs were segregated from the supernatant by centrifugation at 10,000 rpm for 2 h in Beckman L8-70M Ultracentrifuge and SW41 rotor (Beckman Coulter, Brea, CA, USA) at 4 °C through 1 mL of 10% sucrose. The VLP pellet was resuspended in PBS and stored at 4 °C.

### 2.2. Immunoblotting Gel Analysis

The VLPs are diluted in PBS from the stock solution in the ratio 1:1. The VLPs were treated with heterodimerizer HAXS8 (from Tocris Biosciences, Bristol, UK) at a concentration of 1 μM and incubated for the indicated time at 37 °C. They were denatured immediately by Lamelli sample buffer (Bio-Rad Laboratories, Hercules, CA, USA) with 5% BME. The samples were boiled at 95 °C for 5 min. The samples were loaded and proteins were separated by SDS-PAGE. The gels were transferred to a PVDF membrane (Millipore, Burlington, MA, USA). Membranes were washed with blocking buffer and then stained with anti-HIV-1 p24 monoclonal (183-H12-5C; National Institutes of Health, AIDS Reagent Program, National Institutes of Health, Bethesda, MD, USA) primary antibody overnight and then immunoprobed with appropriate infrared anti-mouse secondary antibody for 45 min. The membrane was scanned with the Odyssey Infrared Imaging System (LI-COR Biosciences, Lincoln, NE, USA) according to the manufacturer’s manual instruction at 700 nm. To observe the effect of melittin on the VLPs using immunoblotting gel analysis the VLPs were treated with 0.2 μM of melittin (M2272, from Sigma-Aldrich, St. Louis, MO, USA) and incubated at 37 °C for 15 min before addition of HAXS8 (at a concentration of 1 μM) and then continued with the above outlined protocol. The amount of proteins in each band was quantified by analyzing the cumulative intensity of each band using ImageJ software. That 50% of the fluorescent protein would not be functional was taken into consideration while quantifying the number of proteins in the bands that had proteins that were tagged with either SNAP or Halo.

### 2.3. Simulations for Modeling the Lattice Dynamics

A 2-dimensional hexagonal lattice with a size of 8 nm and ~5000 lattice points were generated. The lattice sites were randomly populated with 2000 Gag molecules. The initialization was biased to one part of the lattice such that the other part of the lattice is completely empty, to replicate the void space present in a HIV lattice. In one of the models 10% of the total molecules were tagged with Halo and the other 10% were tagged with SNAP. In the second model 20% of the total number of molecules were tagged with Halo and the other 20% with SNAP. In the third model we had 40% of the total number of molecules tagged with SNAP and the other 40% with Halo. The molecules hopped on the lattice following a Monte Carlo algorithm and with spherical boundary conditions imposed. The move is rejected if three molecules already occupy the nearest site. Out of the three available neighboring sites the molecule chose its moving site based on an unbiased random number (rand in MATLAB). No binding interactions between the Gag molecules has been taken into consideration except when a Gag-SNAP and Gag-Halo met on a lattice site. It was then treated as a covalently bonded complex, and they hopped as a single entity but always considered to be 2 molecules. The move of a Gag-SNAP-Halo-Gag was allowed to the nearest neighbor provided they did not infract the rule that a lattice site cannot be occupied by more than 3 molecules. The number of Gag-SNAP-Halo-Gag complexes was observed over time. The hopping in each of the simulations was performed for 1000 frames. The timeframe in the simulations were scaled to real experimental time using Hill’s equation:

(1) $$y=\frac{V_{max}{\left(\alpha x\right)}^{n}}{k^{n}+{\left(\alpha x\right)}^{n}}$$

where *y* is the percentage of complex formed over time (*x*), *α* reports the pseudo diffusion coefficient, $V_{max}$, *k* and *n* are scaling factors that solely depends on the percentage of Gag molecules that has been tagged with SNAP or Halo. The value of *α* was fixed to 1 in the simulation data to obtain the values $V_{max}$, *k* and *n*. Then these values were fed in the experimental data to obtain the value of the pseudo diffusion coefficient *α*.

The simulation code was written in MATLAB and performed on the compute nodes with two Intel Xeon Gold 6130 CPUs, 32 CPU cores and 96 GB of RAM per node (CHPC, University of Utah, Salt Lake City, UT, USA). The code has been attached in the supplementary materials. The fitting of the simulation and experimental data was performed in ORIGIN (Originlab Corporation, Northampton, MA, USA) using the Levenberg-Marquardt algorithm.

### 2.4. Imaging Using iPALM

A 25 mm #1.5 glass coverslip (from Hestzig, Leesburg, VA, USA) and a 18 mm #1.5 glass coverslip (from FisherBrand, Houston, TX, USA) were used to prepare the sandwich for imaging. The glass coverslips were washed with 70% ethanol and then in 1 M NaOH for 20 min. They were then thoroughly washed in miliQ water and blow-dried with ultra-high-pressure nitrogen. The coverslips were plasma cleaned for 10 min. The 25 mm coverslip was coated with poly-D-lysine from Sigma-Aldrich at a concentration of 50 mg/mL and incubated in a humid atmosphere for 45 min. After the incubation period, the coverslip was thoroughly washed with mQ water and blow-dried with ultra high-pressure nitrogen. The VLPs were then added to the 25 mm coverslip and incubated in a humid atmosphere for 30 min. The coverslip was then washed gently with PBS so as to remove the VLPs that were not immobilized on the coverslip. This 25 mm coverslip was then sandwiched with the 18 mm coverslip and sealed with Clear Weld quick setting epoxy (by J-B Weld) before imaging. For fixation of the VLPs, freshly prepared disuccinimidyl suberate (DSS) (abcam, Cambridge, UK) was added to the VLPs to a final concentration of 5 mM and incubated at room temperature for 30 min. Then, a quench solution (1 M Tris (pH 7.5)) was added to a final concentration of 25 mM to quench the reaction and incubated at room temperature for 1 h before the fixed VLPs were immobilized on the 25 mm coverslip with the same protocol outlined above.

The VLPs were visualized with iPALM on a prototype setup by Thermo Fisher Scientific. The sandwiched sample was secured onto a micro-positioning stage and illuminated by a 315 mW 561 nm laser. Two 60× Apo TIRF objectives (Nikon, Minato city, Tokyo, Japan) focused on the sample from top and bottom. The custom three-way beam splitter was adjusted to get the interference and maintain a 120° phase difference between the 3 cameras (ORCA-Flash 4.0 sCMOS, Hamamatsu, Hamamatsu city, Shizuoka, Japan). The system was calibrated focusing the gold fiducials that are embedded in the 25 mm coverslip so as to get a resolution below 10 nm in both optical and axial directions. The sample was illuminated using a 561 nm laser in a pseudo TIRF condition so that the entire VLP was illuminated uniformly. The Dendra2 molecules, used as a tag for the HIV Gag, were activated by a 405 nm laser. To ensure uniform activation and avoid multiple activations at the same time point the exposure of the 405 nm laser was increased progressively.

**Figure 1:**
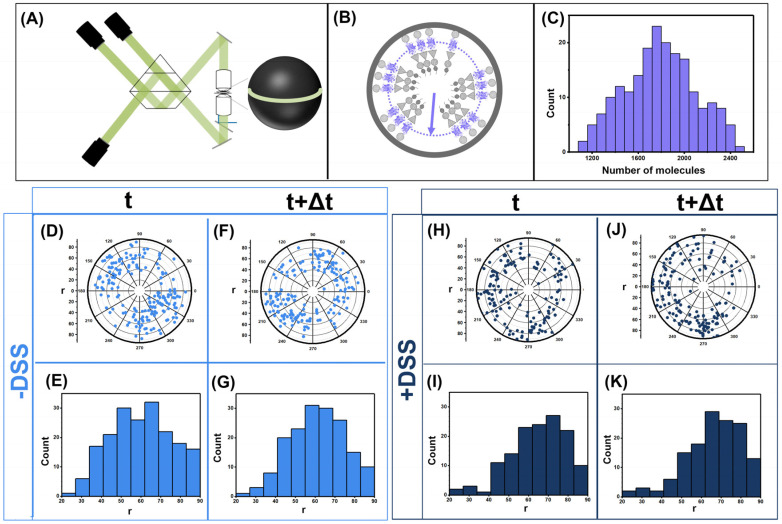
Dynamics of Gag molecules observed by iPALM. HIV Gag(MA-Dendra2-CA) VLPs were produced in HEK293 cells and imaged by iPALM as outlined in text. (**A**) Diagram representation of iPALM. The Gag VLP is shown as a dark sphere, iPALM has 8 nm voxel resolution which allows localization of molecules within the VLP. A 20 nm cross section is shown in green for scale. (**B**) Diagram representation of the position of Dendra2 molecules within the presumed Gag(MA-Dendra2-CA) lattice within the VLP. (**C**) Histogram representation of number of Gag molecules counted in 200 representative VLPs. (**D**–**G**) shows the position of individual Dendra2 molecules in two consecutive localizations within the same VLP (ΔT is 45 min). Significant re-arrangements are visible within the lattice when (**D**,**F**) are compared. (**E**,**G**) show a histogram of the radial position of each Dendra2 molecule localized within the VLP shown in (**D**,**F**) with respect to the center of the VLP (demonstrated by arrow in panel B). (**H**–**K**) show a similar VLP as in (**D**–**G**), except in the presence of DSS which is a membrane permeable cross linker. Significant lower dynamics are observed in (**H**,**J**) in the presence of DSS.

The HIV Gag was tagged with Dendra2 in between MA and CA domain. The VLPs were harvested from 293 T cells as per the protocol described in materials and methods. During the imaging of the HIV GagDendra2 lattice using iPALM (experimental setup shown in Figure 1A), care was taken to photoswitch maximum number of Dendra2 molecules and avoid photoswitching multiple molecules simultaneously. This was successfully achieved by setting the exposure time for the 561 nm laser for 100 ms and the exposure for 405 nm laser was progressively increased form 5 ms exposure every 10 frames to 80 ms exposure every 10 frames. A total of 21,000 frames were collected where the first 1000 frames had no exposure of 405 nm laser. As shown in Figure 1C we have counted a range of 1200 to 2400 molecules within the HIV Gag(MA-Dendra2-CA) VLPs with an average of ~2000. The single molecule counting algorithm has been thoroughly developed in [27]. VLPs with and without fixative were imaged using the same protocol as described.

### 2.5. Cryotomography

Two to 3.5 microliters of purified HIV(D25N) and Gag VLP particles were applied to a thin (2 nm) carbon surface supported by thicker holey carbon (2 µm holes 1 µm apart) and a copper mesh grid (Ted Pella, Redding, CA, USA; Quantifoil Micro Tools GmbH, Großlöbichau, Germany). The specimen was blotted to make thin and plunged into liquid ethane. A Vitrobot (ThermoFisher) was used to prepare these cryogenic specimens. Next, cryo-specimens were imaged in a Titan Krios transmission electron microscope (ThermoFisher) equipped with a Gatan BioQuantum K3 energy filter and direct electron detector. Tilt series from −60° to +60° were recorded at 3° steps over holes in the carbon film via SerialEM software [28]. The microscope was operated at 300 kV with images having a pixel size corresponding to 2.7 Å at the specimen. Slit width of the energy filter was 30 eV. Tilt series were recorded at a range of 1.5–7 µm underfocus. Total electron dose at the specimen was 100–125 electrons per square Å. Three-dimensional reconstructions were computed from the −7 µm defocus tilt series by means of the IMOD software package [29].

### 2.6. Cryotomography Image Reconstruction

Three-dimensional reconstructions were computed from the −7 µm defocus tilt series by means of the IMOD software package [29]. Images were aligned using patch tracking algorithm with more than nine patches used for each tilt series, the software was run using local alignments and residual weighting to achieve optimal alignment with residuals below 6 angstroms as reported by etomo. The aligned series were reconstructed in 512 slices. VLPs and virions were identified in the reconstructed sections and exported as TIF series into ImageJ software. Differential-axis binning (2 pixels in x, 2 pixels in y, and 30 pixels in z) was performed which corresponds to (5.4 Å, 5.4 Å, and 80 Å, respectively) on the TIF slices followed by application of a bandpass filter (specific for highlighting features between 3 and 40 pixels). Mid-sections for each particle were then identified.

### 2.7. Calculating Radial Density in Cryotomography

Central sections of cryotomography reconstructions were analyzed using circular Hough transform to fit the exterior circumference of each particle’s image. By nature of Hough transform, such fits favored regions of higher ordering of the Gag lattice [16]. Radial cross-sections for all particles were taken at pi/32 increments. Cross-sections for regions of high lattice order (prominent EM density variation for membrane/MA domain region) were retained. All sections were aligned at the peak corresponding to membrane density, then averaged and normalized.

## 3. Results

### 3.1. Gag Dynamics Detected Using iPALM Imaging

iPALM microscopy combines the stochastic activation and localization of single fluorescent proteins with an interferometric detection path which allows localization of single molecules with 8 nm resolution in all three dimensions [30]. Given the enhanced resolution of iPALM, it is uniquely suited for imaging molecules in enveloped viruses [31,32]. Our lab has utilized iPALM to image the immature lattice of HIV [26] as well as develop methods to visualize dynamics within this lattice [27]. In our previous studies, we visualized the Gag lattice by fusing photo-switchable fluorescent protein Dendra2 after the p6 domain of Gag. HIV virions have a diameter of ~140–160 nm. By positioning the Dendra2 molecules after Gag p6, we observed a radial distance of ~30 nm for Dendra2 molecules when measured with respect to the center of the VLP, consistent with the expected position of Dendra2 [27].

One simple way to improve our resolution of localizing Gag molecules within the VLP, was to move the Dendra2 molecules further towards the membrane, allowing localization of molecules on a sphere with a larger radial distance. In this study, therefore, we designed a Gag construct by inserting Dendra2 in between the MA and CA domains of Gag, Gag(MA-Dendra2-CA), utilizing similar design as the iGFP HIV backbones [33] as detailed in supplementary material. These constructs retain all domains of Gag including p6. To further characterize the VLPs we followed a protocol developed by Dettenhofer et al. [34]. Highly purified Gag(MA-Dendra2-CA) VLPs were observed at similar densities as HIV(D25N) in a 20–60% gradient spin as shown in the supplementary materials.

Purified Gag(MA-Dendra2-CA) VLPs were immobilized on glass coverslips and imaged in iPALM as previously described in [27] and in methods. Briefly, 21,000 frames were acquired from the sample with a rate of 100 mSec/frame which results in an experiment that is ~90 min long. Using periodic 405 nm excitation, we were able to photoswitch the Dendra2 molecules sequentially and localize ~1200 Dendra2 molecules/VLP. Our previous characterization of Dendra2 has shown that 40% of Dendra2 molecules never photoswitches and therefore 1200 copies of Dendra2 molecules will correspond to ~2000 copies of Gag(MA-Dendra2-CA) molecules in each VLP [27]. Representative results from 200 VLPs analyzed with iPALM are shown in Figure 1.

Figure 1A,B shows a diagram of the iPALM instrument as well as expected organization of Gag(MA-Dendra2-CA) within individual Gag(MA-Dendra2-CA) VLPs. The measured number of Gag molecules/VLP is shown for 200 VLPs in Figure 1C. As shown, there is a distribution between 1200–2400 Gag molecules/VLP with an average of ~2000 Gag molecules within each VLP. To observe dynamics within each VLP, a 16 nm slice of the Gag-Dendra2 localizations is shown in Figure 1D,F. The localizations shown in Figure 1D,F are from the same VLP imaged for a total of 90 min. Localizations in D are all within the initial phase of the imaging that is ~45 min long followed by localizations in F. The nature of iPALM imaging relies on photobleaching each Dendra2 molecule after localization. Therefore, naturally none of the Dendra2 localizations observed in Figure 1D are precisely re-localized in Figure 1F; however, if there was a patch of Gag lattice, we would expect in general many molecules localizing within the same patch in both D&F. We have previously worked out a statistical model to deduce the probability of having the molecules localized in D&F being from the same patch of Gag lattice [27]. If we detect an average of n molecules within a given area at a time point t and n’ molecules at a time point t+Δt. Then the probability whether the structure is retained can be calculated by $P=e^{-{\left(\frac{n^{\prime }-n}{\sigma }\right)}^{2}}$, where σ=√n. This analysis shows that the probability that the structure is retained for the VLPs without fixative (D & F) is $e^{-16}<P<0.3$ whereas for the VLPs with fixative (H & J) the probability of the structure retention is 0.6<P<1. We have previously argued that this analysis is consistent with significant lattice re-organizations within the VLP [27].

To verify the dynamics observed with iPALM, we have imaged Gag(MA-Dendra2-CA) in the presence of a membrane permeable fixative (DSS) as described in methods. Figure 1H,J show the iPALM dynamics observed in VLPs fixed by DSS. This time, statistical analysis of the data shown between H&J shows that its very likely, with a probability of ~0.9 that the localizations are corresponding from the same patch of Gag(MA-Dendra2-CA) lattice within the VLP. As expected by our design of Gag(MA-Dendra-2-CA) constructs, the average distance between Dendra2 localizations and the center of the VLP was ~60 nm which is consistent with having Dendra2 localized below the membrane of the ~140–160 nm HIV virions.

As evident from this section, iPALM analysis is more complex than simple imaging approaches and heavily dependent on statistics for proper analysis. To have enough localizations to decipher a patch of Gag molecules and maintain temporal distance between localizations required for individual single molecule localizations iPALM experiments take ~90 min and can generate approximately two frames capturing the statistical localization of Dendra2 molecules within the cavity. While this approach is sufficient for a statistical estimate of presence of dynamics within the Gag(MA-Dendra2-CA) lattice, it cannot quantify the rate of Gag(MA-Dendra2-CA) dynamics within the VLP.

### 3.2. Gag Dynamics Detected Using Kinetic Biochemical Assay

If Gag molecules are undergoing significant dynamics within the Gag VLPs, as is suggested by our data presented in Figure 1 for Gag(MA-Dendra2-CA) VLPs and our previous measurements of Gag-Dendra2 VLPs in [27], these dynamics should have detectable biochemical implications. Given that all iPALM data is generated using fluorescent protein inserts within Gag molecules, developing a biochemical assay also has the additional advantage of reduced perturbation of Gag within the VLPs.

**Figure 2:**
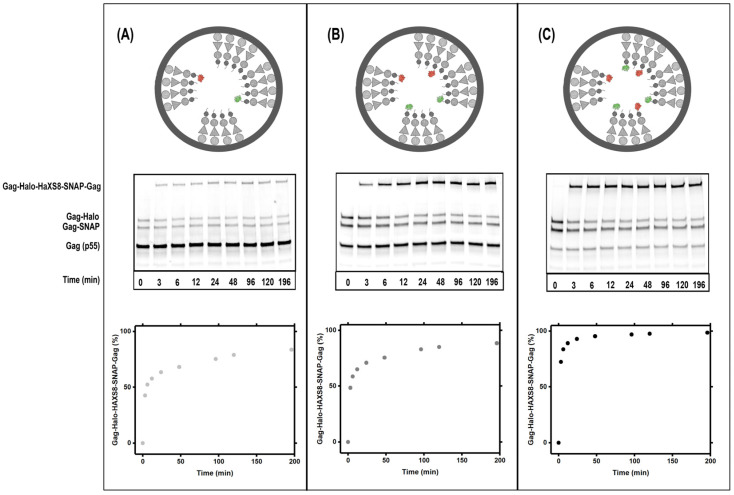
Dynamics detected by kinetic biochemical assay. HIV Gag VLPs were assembled from HEK293 cells by expressing: (**A**) Top shows a model of a Gag VLP with 80% Gag, 10% Gag-SNAP and 10% Gag-Halo. Both Halo and SNAP are fused to Gag after the p6 domain as further explained in text. HaXS8 is applied to the VLP solution at time zero and VLP fractions are harvested according to the displayed time. The ratio of the hetero-dimer formation in the presence of HaXS8 as a function of time is quantified from the western blots. (**B**) Similar to (**A**) except with 60% Gag, 20% Gag-SNAP and 20% Gag-Halo. (**C**) similar to A and B except with 20% Gag, 40% Gag-SNAP and 40% Gag-Halo.

We have previously developed a biochemical kinetic assay to measure dynamics within the Gag(VLPs) through measurements of hetero-dimerization of a small fraction of Gag-SNAP and Gag-Halo molecules added to otherwise untagged Gag within Gag VLPs [27]. SNAP and Halo proteins form covalently bound heterodimers in the presence of the membrane permeable HaXS8 crosslinker [35,36]. In experiments presented in Figure 2, we show the dynamics of Gag-SNAP-Gag-Halo heterodimer formation within VLPs incorporating a different fraction of Gag-SNAP and Gag-Halo molecules. Specifically Figure 2A shows dynamics in VLPs with 80% Gag, 10% Gag-SNAP and 10% Gag-Halo proteins. Figure 2B shows dynamics in VLPs with 60% Gag, 20% Gag-SNAP and 20% Gag-Halo proteins. Figure 2C shows dynamics in VLPs with 20% Gag, 40% Gag-SNAP and 40% Gag-Halo proteins.

As intuitively expected, increasing the percentage of Gag-SNAP and Gag-Halo within the VLP results in faster kinetics for hetero-dimer formation. The biochemical analysis has the advantage of providing multiple measurements in the 0–200-min span. Since the western blots are performed on a pool of VLPs simultaneously; the results are reproducible and show kinetics of the hetero-dimer formation from 0–200 min. While, from observations of the raw data presented in Figure 2, one can argue that major lattice re-organizations are happening within first 30-min timeframe, a more accurate mathematical model needs to be developed for analysis of the data.

### 3.3. Quantifying Gag Dynamics by Monte-Carlo Simulations

**Figure 3:**
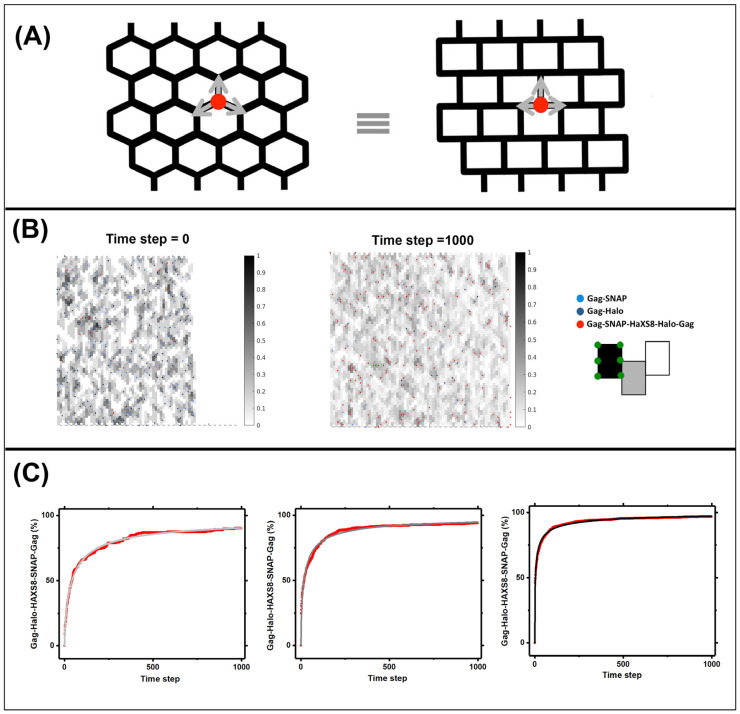
Monte Carlo model of Gag dynamics. In this model we assume that the full surface of the VLP is covered by a lattice as shown in (**A**) Gag molecules are initialized onto this lattice at time point zero and occupy 80% of the lattice. At each subsequent time interval, each Gag molecule is moved according to its position on the lattice and occupancy state of the neighboring vertexes. There was no barrier for leaving a vertex and any vertex could be entered upon as long as it had a lower occupancy than three Gag molecules. (**B**) When a Gag-SNAP and a Gag-Halo molecule occupy the same vertex, they will bind to each other and form a heterodimer. The hetero dimers remain bound to the end of the simulation. (**C**) Percentage of hetero-dimer formation in the simulations with 10%, 20% and 40% Gag-SNAP and Gag-Halo proteins were fitted to the mathematical model presented in the text and shown in red.

In principle, Gag dynamics within the VLPs can be analyzed using molecular dynamics simulations which can take into account all known interactions of Gag with both membrane and other Gag molecules. However, such a detailed model is computationally expensive and therefore cannot be used easily to model the experimental biochemical data presented in Figure 2. The data presented in Figure 2, while highly reproducible, does not contain sufficient information to decipher multiple parameters of any model independently. We therefore created a MonteCarlo model with significant simplifications of Gag interactions within the VLP. In our model which is described in detail within the methods section, Gag molecules walk on a 2D hexagonal lattice which covers a surface area similar to the area inside the VLP with spherical boundary conditions. In this model, the Gag molecules do not experience any Gag-Gag interactions. Their only interaction with other Gag molecules occurs when three Gag molecules occupy a vertex in the lattice, blocking the addition of any other Gag molecules to that vertex. This deliberate assumption has been made to reduce the number of free parameters within the model; therefore in reality, the pseudo-diffusion reported by our model is a complex superposition of all biochemical interactions experienced by Gag molecules and their free diffusion along the internal leaflet of the viral membrane. Figure 3 shows the results of these MonteCarlo simulations in lattices incorporating 10%, 20% or 40% Gag-SNAP and Gag-Halo molecules.

Figure 3A shows the hexagonal lattice within the MonteCarlo simulations. In each time step, every Gag molecule within the lattice moves along the available lattice directions shown with grey arrows to reach its nearest neighbors. The movement is successful only if the new destination is not already occupied by three Gag molecules. The movements are repeated for 1000 steps as shown in Figure 3B keeping track of every Gag molecule in the extended VLP lattice. Gag-SNAP and Gag-Halo molecules that occupy the same vertex at any given time, are permanently bound with each other and further diffuse as a single unit. Figure 3C shows the accumulation of Gag-SNAP, Gag-Halo heterodimers in the VLPs as a function of time. The resulting shape of the curves is similar to the data obtained from experiments shown in Figure 2, with one significant difference. While data in Figure 2 is plotted versus minutes, data from the Monte Carlo simulations is plotted versus frame number. This is by design, since Monte Carlo simulations are performed on a frame-by-frame basis. If the Monte Carlo simulations are scaled to exactly fit the experimental data, then the time step of the Monte Carlo simulations Tm can be set to real time in minutes. Since every Gag molecule travels one length of the hexagonal lattice in each Monte Carlo step, a pseudo diffusion coefficient can be measured as $D_{pseudo}=\frac{{\left(8\;\mathrm{nm}\right)}^{2}}{T_{m}}$. To estimate $T_{m}$ we used the equation derived in methods to parametrize the results of the Monte Carlo simulations as shown in Figure 3C with red curves. These parametrized curves are then used to fit the real experimental data and obtain the $T_{m}$.

### 3.4. Gag Dynamics Are Sensitive to Mutations within SP1 Region and Abrogated with Melittin

**Figure 4:**
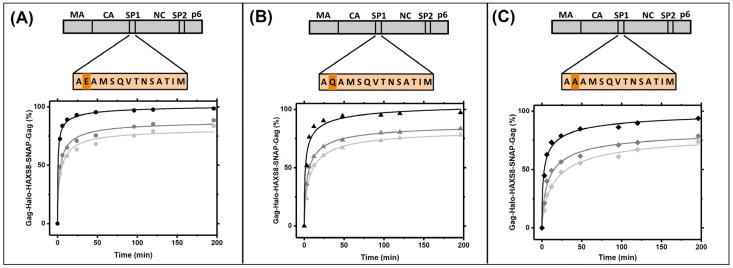
Quantification of Gag diffusion in SP1 mutants. The mathematical model developed in Figure 3 is applied to the raw data from (**A**) WT, which result in a pseudo diffusion coefficient of 10 nm^2^/s (**B**) E2Q which has 5 nm^2^/s and (**C**) E2A mutant which has a pseudo diffusion coefficient of 2 nm^2^/s.

Having established a Monte Carlo model to analyze the biochemical kinetic hetero-dimerization curves, we performed the hetero-dimerization experiments at three different concentrations of Gag-SNAP and Gag-Halo incorporation (10, 20 and 40%) in VLPs created by expressing WT Gag in Figure 4A, Gag(E2A) in Figure 4B and Gag(E2Q) presented in Figure 4C. Both E2A and E2Q mutations have been originally identified as part of studies implicating the importance of SP1 helix formation for maturation of HIV [21,37]. Assembly, release and maturation of HIV are tightly linked and therefore, we aimed to choose mutants which had a subtle effect on infectivity to see if we can detect any changes in the lattice dynamics. As is shown in [37], while particle morphologies and release efficiencies are not affected, propagation of these mutants in Jurkat cells is slowed in the case of E2Q and severely limited in the case of E2A. The sequence of Amino acids within each construct is shown in Figure 4. Solid lines within the figure represent scaled Monte Carlo models fitted to the experimental data to obtain the T_m_ parameter for each condition. Using these fits, we report pseudo diffusion coefficients of ~10 nm^2^/s for WT Gag, ~4 nm^2^/s for E2Q and ~2 nm^2^/s for E2A mutations. Specifically, the E2A mutant is expected to increase the probability of helix formation within the SP1 domain, which should in principle increase the avidity of Gag within the Gag lattice inside VLPs.

**Figure 5:**
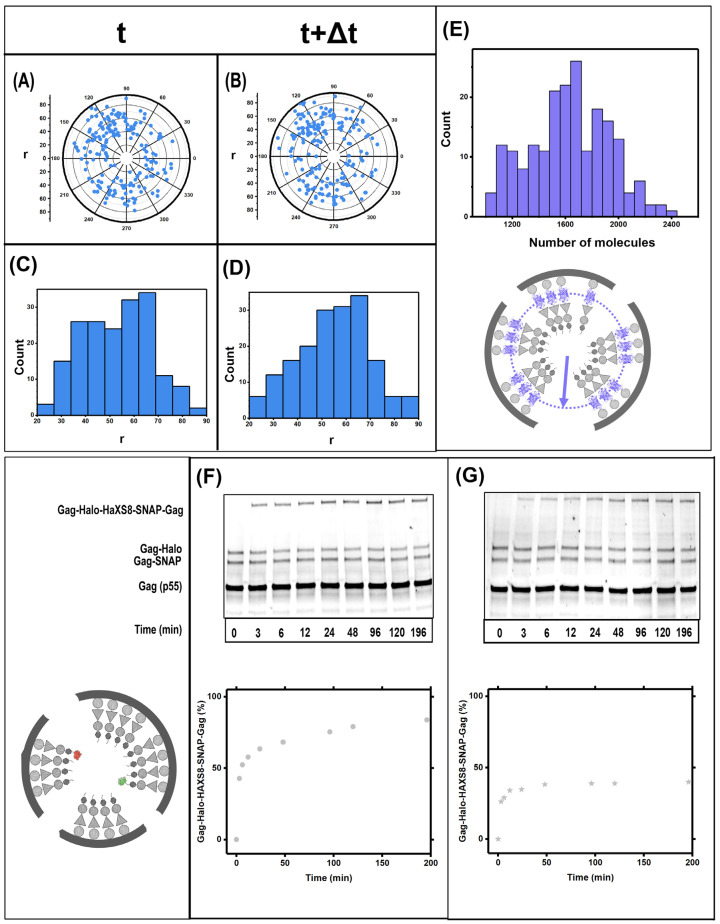
Effects of melittin on Gag dynamics within VLPs. (**A**–**E**) shows the measurements of dynamics in Gag(MA-Dendra2-CA)VLPs utilizing iPALM as previously shown in Figure 1. As shown in (**C**,**D**) We detect a slight decrease in the radius of the Dendra2 localization within the VLPs punctuated by melittin when compared to the VLPs shown in Figure 1. The number of Gag molecules within 200 representative VLPs is shown in € and the histogram is similar to the histogram of number of Gag molecule s presented in Figure 1. Therefore addition of melittin did not result in loss of Gag molecules from the VLPs. There is no dynamic detected within the VLP as shown in A and B in the presence of melittin. (**F**,**G**) show the rate of formation of Gag-Snap-Gag-Halo hetero dimer in the presence of HaXS8 and different concentration of melittin (**F**) 0.1 μM and (**G**) 0.2 μM.

If Gag molecules are indeed moving within the interior of the VLP, given the strong MA domain membrane interactions that recruit Gag to bud from the plasma membrane of the infected cells, one would expect that all Gag molecules within the VLP should be anchored within the membrane through their MA interactions. This is supported by the measured radial distribution of Dendra2 molecules as presented in Figure 1 using iPALM. Any re-organization of Gag therefore should be limited to 2D diffusion along the inner leaflet of the virion membrane. Indeed both our iPALM data as well as cryotomography demonstrate a significant portion of the VLP surface to be void of the Gag lattice [16,26]. It is therefore reasonable to hypothesize that this free membrane is essential for diffusion of Gag molecules within the VLP. To partially test this hypothesis, we utilized melittin which has been used previously to poke holes within the membrane of HIV for purification of mature capsids [38]. Figure 5 shows how the application of melittin to the purified VLPs have affected the dynamics of Gag within VLPs.

The effects of melittin were first quantified on the dynamics of Gag(MA-Dendra-CA) within Gag(MA-Dendra-CA) VLPs. Figure 5E shows the distribution of the number of Gag(MA-Dendra-CA) molecules within VLPs treated by 0.2 μM of melittin. As shown in Figure 5E, there are 1200–2400 Gag molecules incorporated in each VLP which is similar to VLPs not treated with melittin as shown in Figure 1. The Dendra2 molecules are also localized at an average distance of 60 nm from the VLP center, which is consistent with the VLPs retaining their structural integrity while under the treatment with melittin. Figure 5A,B show two consecutive reconstructions with an approximately 45 min temporal separation. Statistical analysis of the localized Dendra2 molecules in Figure 5A,B shows with ~60% probability that all molecules emanate from the same mass of Gag molecules within the VLP with no detectable dynamics. The dynamics of Gag within VLPs in the presence of melittin was further assayed in the presence of 0.1 μM and 0.2 μM melittin as presented in Figure 5F,G. Similar to results reported by iPALM, the 0.2 μM melittin abrogated the dynamics within the VLPs measured in the biochemical heterodimerization assay. We therefore conclude that the presence of membrane, is essential for diffusion of Gag within the VLPs.

### 3.5. Cryotomography of Immature HIV and Gag VLPs

**Figure 6:**
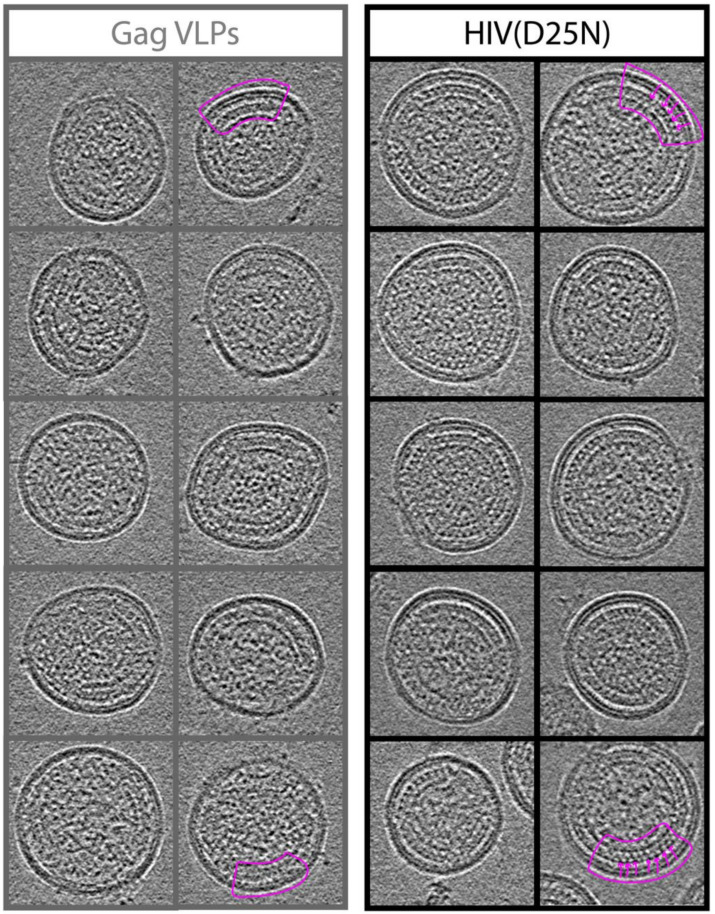
Cryotomography of HIV(D25N) and Gag VLPs. Mid-sections from cryotomography reconstructions are shown for 10 HIV(D25N) and Gag VLPs. In some panels, the Gag lattice is identified with the pink contour in both Gag VLPs as well as HIV (D25N) virions. Pink arrows show the striated density expected from the CA-CTD-SP1 folding which is prominent in the HIV(D25N) virions and not observed in Gag VLPs with the same prominence.

As data presented in previous sections demonstrates, there is clear evidence of Gag dynamics within Gag VLPs. The rate of $10{\;\mathrm{nm}}^{2}/\sec$ measured for pseudo diffusion of Gag within the VLP is significant since it would mean, on average, each Gag molecule would jump to the next vertex in a hexagonal lattice within a second. This rate is not commensurate with data from cryotomography measurements, which show each Gag molecule interacting with at least five other Gag molecules within the lattice. Up to this point, we had assumed, partially because of the available biochemical evidence as well as the abundance of Gag-Gag interactions, that VLPs assembled in mammalian cells will form a similar Gag lattice to the one observed in immature HIV virions. This assumption is shared to large extent with all other groups which had characterized Gag VLPs using TEM thin sections and negative staining protocols [25]. To test this assumption, we produced highly purified immature HIV virions by expressing the NL4.3(D25N) backbone, or Gag VLPs by expressing HIV Gag in HEK293 cells and following the steps suggested by Dettenhofer et al. [32] to create highly purified virions for cryotomography analysis as shown in Figure S1. Representative reconstructions are shown in Figure 6 for both HIV(D25N) as well as Gag VLPs.

Figure 6 shows ten representative reconstructions of Gag VLPs as well as ten HIV(D25N) immature virions. A Gag lattice is identified in both HIV(D25N) as well as Gag VLPs highlighted by the pink contours in the images. HIV(D25N) virions also show a characteristic ordering of the CA-CTD and SP1 region within the Gag lattice [16,17,19] as is highlighted by pink arrows in Figure 6. From the 10 HIV(D25N) particles shown in the figure, nine virions show clear structures corresponding to the ordering observed for the CA-CTD and SP1 structures. In contrast, from the 10 Gag VLPs shown, it is clear that the Gag lattice, while still detectable within these VLPs, does not resemble the full order observed in the HIV(D25N) virions. Specifically, nine out of ten reconstructions completely lack the characteristic CA-CTD and SP1 ordering observed in the HIV(D25N) virions. We therefore conclude that the Gag ordering within the VLPs is different from the ordering observed in the HIV(D25N) virions.

**Figure 7:**
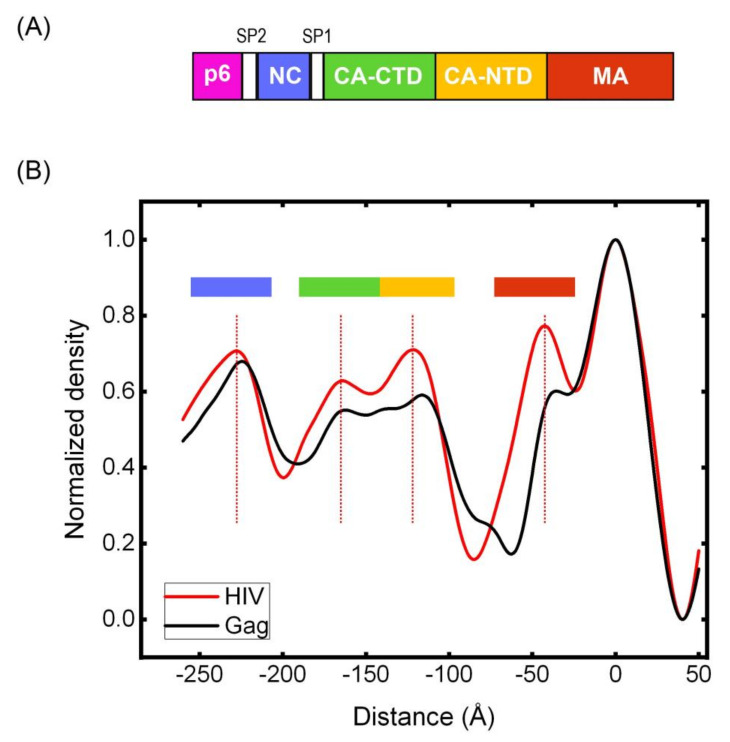
Radial density plots of HIV(D25N) and Gag VLPs. Average radial densities calculated for HIV(D25N) and Gag(VLPs) shown in Figure 6. (**A**) Shows a diagram of Gag domain structures and (**B**) shows the radial densities with their corresponding domains.

We have further calculated the radial density function for HIV(D25N) virions and compared them to Gag VLPs as shown in Figure 7. All major domains of Gag including MA, CA-NTD, CA-CTD and NC are clearly identifiable in both radial densities; however, the radial densities show significant loss of ordering in the CA domain which results in less clear separation between CA-NTD and CA-CTD regions. The average densities for Gag VLP domains are also slightly but consistently shifted towards the membrane along all observed domains.

## 4. Discussion

Here we show significant evidence that Gag molecules assembled into VLPs incorporating similar numbers of Gag molecules to HIV virions, fail to create a stable Gag lattice. Given that significant Gag-Gag interactions have been previously identified, we did not expect the observed results. Here we will first discuss all experimental conditions followed by possible ramifications of our observed results.

Since Gag-Gag interactions are essential for formation of the immature lattice, we will first discuss all experimental conditions relevant to formation of Gag VLPs. The Gag VLPs used in this study have been highly purified in equilibrium sucrose gradients and float at the same density as immature HIV virions. The counting of Dendra2 molecules within each VLP is methodical and precise [27]. It takes 90 min to interrogate each VLP with iPALM and each Dendra2 molecule is activated and photobleached before all the others are counted. We therefore do not expect large errors in our Dendra2 counting. Although we counted ~2000 copies of Gag in each VLP, the counting in immature HIV virions has been performed by cryotomography [15]. Given the coverage of 30–60% of the virion membrane by the immature Gag lattice inside HIV virions [16], and the estimated 5000 Gag copies required to fully cover the inner surface of the virions [39], the estimated 2000 copies of Gag within each HIV virion seems to be a very reasonable estimate. It is therefore reasonable to assume that both Gag VLPs as well as HIV virions are packaging ~2000 copies of Gag within each VLP. It is always possible that introduction of fluorescent tags can disrupt the Gag lattice, however, the lattice dynamics described in this manuscript are reproduced regardless of the position and density of protein tags which included tagging all Gag molecules between MA and CA domains, adding the tag after the Gag-p6 in all Gag molecules as well as only 10% of Gag molecules. It is therefore unlikely that positions of the tags have significantly affected the Gag dynamics. In this regard, we have performed the Cryotomography presented in Figure 6 and Figure 7 in VLPs devoid of any additional protein tags and observed a significant difference between the lattice of Gag VLPs and HIV(D25N) virions. The lack of structure in the CA-CTD and SP1 regions, would reduce Gag-Gag interactions and can support the observed Gag dynamics observed in Biochemical and iPALM imaging experiments, although it is important to point out that this would only be a suggestion since we currently lack methods to detect dynamics without introduction of protein tags.

We have used Monte-Carlo simulations to obtain pseudo-diffusion of Gag molecules within isolated VLPs. A full model of Gag dynamics requires parameters describing all of the Gag interactions within the immature lattice (“on” and “off” rates) and the varied mechanisms of diffusion of Gag. Such a model would have a large number of free parameters and would not be properly constrained with the biochemical data presented in this manuscript. We have therefore introduced the pseudo-diffusion of Gag which is a combination of all “on” and “off” binding rates as well as purely diffusive behavior. The pseudo-diffusion only contains a general description of dynamics within the VLPs. To dissect all the parameters involved in Gag dynamics, additional novel methods for detecting interactions within the VLPs have to be developed.

Incorporation of IP6 has been shown to play a role in the formation of immature HIV lattice [14]. Both the HIV virions as well as Gag VLPs used for cryotomogrtaphy were assembled in HEK293 cells which produce sufficient IP6 levels required for virus budding [13]. It is also very unlikely that Gag VLPs would have had less access to IP6 than HIV virions, however we should note that if there is a mechanism to enhance the incorporation of IP6 in the virions versus Gag VLPs, it could partially explain the observed results and this hypothesis can be explored in future.

While none of the HIV accessory proteins, which include Nef, Vpr, Vif, and GagPol, are expected to have any structural role in the assembly of the immature lattice, they are technically absent during the assembly of Gag VLPs; therefore, one cannot rule out however unlikely at least an indirect role for these proteins in the structural stability of the immature lattice. Incorporation of the ENV protein within the envelope of the immature HIV virions, was shown to affect the overall stiff ness of the immature virions as measured by atomic force microscopy [40] The increase in stiffness is specific to the immature virions and becomes negligible after maturation. The cytosolic domain of HIV ENV interacts with the immature HIV Gag lattice and play a role in recruitment of ENV into budding HIV virions [32,41,42]. What if any effect these interactions have on the stability of the immature lattice will remain to be determined?

The main difference between the HIV virions and Gag VLPs, however, is the incorporation of the gRNA in the immature Gag lattice of the HIV virions. While the full picture of the gRNA interaction with Gag is very complex, it has been shown using crosslinking-immunoprecipitation (CLIP) sequencing that gRNA interacts with Gag along all its length within the immature HIV virions [43] and Gag molecules dimerize on the gRNA packaging signal [44]. It has also been suggested a while back that gRNA plays a structural role in the immature HIV virions [45]. It is therefore a possibility that gRNA interactions are what stabilize the immature Gag lattice within the HIV virions. Further experiments would be required to test this hypothesis.

Previous comparisons between HIV Gag and other retroviral Gag VLPs have been carried out using both negative staining as well as cryotomography [46,47]. The significant difference observed in our cryotomography between full length immature HIV virions and Gag VLPs were neither explored nor reported before as prior studies had been primarily focused on the shape and curvature of the lattice and not necessary a comparison in ordering of Gag molecules. It is worth pointing out that the reconstructions of Gag VLPs presented in these studies have significant resemblance to our cryotomography from purified VLPs.

A lattice of Gag molecules lining the inner leaflet of the membrane has been identified in all retroviral genera including alpharetroviruses, betaretroviruses, deltaretroviruses, epsilonretroviruses, gammaretroviruses and lentiviruses (which include HIV) [22,46,48]. Recently, it has also been shown that neuronal gene Arc encodes a Gag protein which forms a lattice in neuronal cells and mediates RNA transfer [49]. Our study suggests that in HIV, other elements in addition to Gag are involved in maintaining order within the Gag lattice. How much such additional ordering is essential for the Gag function in other retroviruses or neuronal functions and what other elements are required for maintaining this order will remain to be further investigated.

## Data Availability

All data are available in the main text or the supplementary materials.

## Supplementary Materials

The following are available online at https://www.mdpi.com/article/10.3390/v13101946/s1, Sequences of protein constructs used in this study. Matlab code for MonteCarlo simulation of lattice hopping for 10% tagged molecules. Figure S1. Fractions from 20–60% sucrose gradient of HIV(D25N), Gag(MA-Dendra2-CA) and Gag(WT) VLPs analyzed by protein stain. Density of each fraction is typed on top of each lane. Figure S2. The VLPs were treated with HAXS8 of the indicated concentration and incubated in 33C. (A) shows western blot analysis of VLPs incorporating 80% Gag, 10% Gag-SNAP and 10% Gag-Halo treated with HAXS8 of indicated concentration. (B) shows western blot analysis of VLPs incorporating 60% Gag, 20% Gag-SNAP and 20% Gag-Halo treated with HAXS8 of indicated concentration. (C) shows western blot analysis of VLPs incorporating 20% Gag, 40% Gag-SNAP and 40% Gag-Halo treated with HAXS8 of indicated concentration. (D) indicates the density of SNAP-Halo complex obtained in each case of (A). (E) indicates the density of SNAP-Halo complex obtained in each case of (B). (F) indicates the density of SNAP-Halo complex obtained in each case of (C).
