# Supplementary material for: Gag-Gag Interactions Are Insufficient to Fully Stabilize and Order the Immature HIV Gag Lattice
Source: Viruses. 2021 Sep 28;13(10):1946. doi: 10.3390/v13101946 (PMC8540168; doi:10.3390/v13101946)
Supplement: Supplementary file 1 [file viruses-13-01946-s001.zip › viruses-1385312-supplementary.pdf]

Title

- Gag-Gag interactions are insufficient to fully stabilize and order the immature HIV Gag lattice.

Authors

Ipsita Saha[1], Benjamin Preece[2,3], Abby Peterson[2,3], Haley Durden[2,3], Brian MacArthur[2,3], Jake Lowe[4], David Belnap[4,5], Michael Vershinin[2,3,4] and Saveez Saffarian[2,3,4,*]

Affiliations

- [1] Laboratory of Cell and Developmental Signaling, Center for Cancer Research, National Cancer Institute, National Institutes of Health
- [2] Center for Cell and Genome Science, University of Utah
- [3] Department of Physics and Astronomy, University of Utah
- [4] School of Biological Sciences, University of Utah
- [5] Department of Biochemistry, University of Utah
- * Corresponding author: Saffarian@physics.utah.edu

Sequences of protein constructs used in this study:

Gag:
MGARASVLSGGELDRWEKIRLRPGGKKKYKLKHIVWASRELERFAVNPGLLETSEGCR
QILGQLQPSLQTGSEELRSLYNTVATLYCVHQRIEIKDTKEALDKIEEEQNKSKKKAQQA
AADTGHSNQVSQNYPIVQNIQGQMVHQAISPRTLNAWVKVVEEKAFSPEVIPMFSALSE
GATPQDLNTMLNTVGGHQAAMQMLKETINEEAAEWDRVHPVHAGPIAPGQMREPRGS
DIAGTTSTSTLQEQIGWMTHNPPIPVGEIYKRWIILGLNKIVRMYSPTSILDIRQGPKEPFR
DYVDRFYKTLRAEQASQEVKNWMTETLLVQNANPDCKTILKALGPGATLEEMMTACQ
GVGGPGHKARVLAEAMSQVTNPATIMIQKGNFRNQRKTVKCFNCGKEGHIAKNCRAPR
KKGCWKCGKEGHQMKDCTERQANFLGKIWPSHKGRPGNFLQSRPEPTAPPEESFRFGE
ETTTPSQKQEPIDKELYPLASLRSLFGSDPSSQ

Gag(MA-Dendra2-CA):
MGARASVLSGGELDRWEKIRLRPGGKKKYKLKHIVWASRELERFAVNPGLLETSEGCR
QILGQLQPSLQTGSEELRSLYNTVATLYCVHQRIEIKDTKEALDKIEEEQNKSKKKAQQA
AADTGHSNQVSQNYPIVQTRMNTPGINLIKEDMRVKVHMEGNVNGHAFVIEGEGKGKP
YEGTQTANLTVKEGAPLPFSYDILTTAVHYGNRVFTKYPEDIPDYFKQSFPEGYSWERT
MTFEDKGICTIRSDISLEGDCFFQNVRFKGTNFPPNGPVMQKKTLKWEPSTEKLHVRDG
LLVGNINMALLLEGGGHYLCDFKTTYKAKKVVQLPDAHFVDHRIEILGNDSDYNKVKL
YEHAVARYSPLPSQVWRSQNYPIVQNIQGQMVHQAISPRTLNAWVKVVEEKAFSPEVIP
MFSALSEGATPQDLNTMLNTVGGHQAAMQMLKETINEEAAEWDRVHPVHAGPIAPGQ
MREPRGSDIAGTTSTSTLQEQIGWMTHNPPIPVGEIYKRWIILGLNKIVRMYSPTSILDIRQ
GPKEPFRDYVDRFYKTLRAEQASQEVKNWMTETLLVQNANPDCKTILKALGPGATLEE
MMTACQGVGGPGHKARVLAEAMSQVTNPATIMIQKGNFRNQRKTVKCFNCGKEGHIA

KNCRAPRKKGCWKCGKEGHQMKDCTERQANFLGKIWPSHKGRPGNFLQSRPEPTAPPE
ESFRFGEETTTPSQKQEPIDKELYPLASLRSLFGSDPSSQ

Gag-SNAP:
MGARASVLSGGELDRWEKIRLRPGGKKKYKLKHIVWASRELERFAVNPGLLETSEGCR
QILGQLQPSLQTGSEELRSLYNTVATLYCVHQRIEIKDTKEALDKIEEEQNKSKKKAQQA
AADTGHSNQVSQNYPIVQNIQGQMVHQAISPRTLNAWVKVVEEKAFSPEVIPMFSALSE
GATPQDLNTMLNTVGGHQAAMQMLKETINEEAAEWDRVHPVHAGPIAPGQMREPRGS
DIAGTTSTSTLQEQIGWMTHNPPIPVGEIYKRWIILGLNKIVRMYSPTSILDIRQGPKEPFR
DYVDRFYKTLRAEQASQEVKNWMTETLLVQNANPDCKTILKALGPGATLEEMMTACQ
GVGGPGHKARVLAEAMSQVTNPATIMIQKGNFRNQRKTVKCFNCGKEGHIAKNCRAPR
KKGCWKCGKEGHQMKDCTERQANFLGKIWPSHKGRPGNFLQSRPEPTAPPEESFRFGE
ETTTPSQKQEPIDKELYPLASLRSLFGSDPSSQGGGGSMDKDCEMKRTTLDSPLGKLELS
GCEQGLHEIKLLGKGTSAADAVEVPAPAAVLGGPEPLMQATAWLNAYFHQPEAIEEFPV
PALHHPVFQQESFTRQVLWKLLKVVKFGEVISYQQLAALAGNPAATAAVKTALSGNPV
PILIPCHRVVSSSGAVGGYEGGLAVKEWLLAHEGHRLGKPGLG

Gag-Halo:

**MGARASVLSGGELDRWEKIRLRPGGKKKYKLKHIVWASRELERFAVNPGLLETSE
GCRQILGQLQPSLQTGSEELRSLYNTVATLYCVHQRIEIKDTKEALDKIEEEQNKSK
KKAQQAAADTGHSNQVSQNYPIVQNIQGQMVHQAISPRTLNAWVKVVEEKAFSPE
VIPMFSALSEGATPQDLNTMLNTVGGHQAAMQMLKETINEEAAEWDRVHPVHAG
PIAPGQMREPRGSDIAGTTSTSTLQEQIGWMTHNPPIPVGEIYKRWIILGLNKIVRM
YSPTSILDIRQGPKEPFRDYVDRFYKTLRAEQASQEVKNWMTETLLVQNANPDCKT
ILKALGPGATLEEMMTACQGVGGPGHKARVLAEAMSQVTNPATIMIQKGNFRNQ
RKTVKCFNCGKEGHIAKNCRAPRKKGCWKCGKEGHQMKDCTERQANFLGKIWP
SHKGRPGNFLQSRPEPTAPPEESFRFGEETTTPSQKQEPIDKELYPLASLRSLFGSDP
SSQGGGGSMAEIGTGFPFDPHYVEVLGERMHYVDVGPRDGTPVLFLHGNPTSSYV
WRNIIPHVAPTHRCIAPDLIGMGKSDKPDLGYFFDDHVRFMDAFIEALGLEEVVLVI
HDWGSALGFHWAKRNPERVKGIAFMEFIRPIPTWDEWPEFARETFQAFRTTDVGR
KLIIDQNVFIEGTLPMGVVRPLTEVEMDHYREPFLNPVDREPLWRFPNELPIAGEPA
NIVALVEEYMDWLHQSPVPKLLFWGTPGVLIPPAEAARLAKSLPNCKAVDIGPGLN
LLQEDNPDLIGSEIARWLSTLEISG**

Matlab code for MonteCarlo simulation of lattice hopping for 10% tagged molecules

```
l=72; %length of lattice
b=72; %width of lattice
time=1000;
area=l*b;

x=linspace(1,l,l);
y=linspace(1,b,b);

lattice(area).x=[];
lattice(area).y=[];
```

```
lattice(area).occu=[];
k=1;
c3=0;
c2=0;

%assign lattice occupancy
for i=1:l
    for j=1:b
        lattice(k).x=i;
        lattice(k).y=j;
        roll=rand;
        if(roll>=0 && roll<=(600/5184))
            lattice(k).occu(1)=3;
            c3=c3+1;
        elseif(roll>(600/5184) && roll<=(1000/5184))
            lattice(k).occu(1)=2;
            c2=c2+1;
        else
            lattice(k).occu(1)=0;
        end
        k=k+1;
    end
end

%particle
npart=2000;
particle(npart).xp=[];
particle(npart).yp=[];
particle(npart).ts=[];
particle(npart).type=[];
particle(npart).parttype=[];
particle(npart).partidx=[];

%initial localization
ct=1;
snap=0;
halo=0;
gag=0;
for i =1:area
    if(ct<=npart)
        if(lattice(i).occu(1)>0)
            k=lattice(i).occu(1);
                for j=1:k
                    particle(ct).xp(1)=lattice(i).x;
                    particle(ct).yp(1)=lattice(i).y;
                    particle(ct).ts(1)=1;
                    roll=rand;
                    if(roll>=0 && roll<=0.1)
                        particle(ct).type=1;
                        snap=snap+1;
                    elseif(roll>0.1 && roll<=0.2)
                        particle(ct).type=2;
                        halo=halo+1;
                    elseif(roll>0.2 && roll<=1)
                        particle(ct).type=3;
                        gag=gag+1;
```

```
            end
            ct=ct+1;
         end
      end
   end
end

npart=gag+snap+halo;
%determine the partners and partner idx
cp=0;
for i=1:npart
   cp=0;
   for j=1:npart
      if(particle(i).xp(1)==particle(j).xp(1) && particle(i).yp(1)==particle(j).yp(1) && j~=i)
         cp=cp+1;
         particle(i).parttype(1,cp)=particle(j).type;
         particle(i).partidx(1,cp)=j;
      end
   end
   if(cp==1)
      particle(i).parttype(1,2)=0;
      particle(i).partidx(1,2)=0;
   elseif(cp==0)
      particle(i).parttype(1,1)=0;
      particle(i).partidx(1,1)=0;
      particle(i).parttype(1,2)=0;
      particle(i).partidx(1,2)=0;
   end
end

%update lattice occupancy
for i=1:area
   lattice(i).occu=0;
   for j=1:npart
      if(lattice(i).x==particle(j).xp(1) && lattice(i).y==particle(j).yp(1))
         lattice(i).occu=lattice(i).occu+1;
      end
   end
end

%random walk
complexct(1:time-1)=0;
for tt=2:time
   row=0;
   cpst_o=0;
   cpst=0;
   %record the ones which are bonded at time t
   for i=1:npart
      if((particle(i).type==1 && particle(i).parttype(tt-1,1)==2) || (particle(i).type==2 &&
particle(i).parttype(tt-1,1)==1))
         row=row+1;
         cpst_o(row,1)=i;
         cpst_o(row,2)=particle(i).partidx(tt-1,1);
      elseif((particle(i).type==1 && particle(i).parttype(tt-1,2)==2) || (particle(i).type==2 &&
particle(i).parttype(tt-1,2)==1))
         row=row+1;
```

```
            cpst_o(row,1)=i;
            cpst_o(row,2)=particle(i).partidx(tt-1,2);
        end
    end

    for j=1:row
        cpst_o(j,:)=sort(cpst_o(j,:));
    end

    row1=row;
    i=1;
    while (i<=row1)
        j=1;
        while (j<= row1 )
            if((cpst_o(i,1)==cpst_o(j,1) || cpst_o(i,1)==cpst_o(j,2) || cpst_o(i,2)==cpst_o(j,1) ||
cpst_o(i,2)==cpst_o(j,2)) && j~=i)
                cpst_o(j,:)=0;
                %row1=row1-1;
                %break;
            end
            j=j+1;
        end
        i=i+1;
        %row1=length(cpst);
    end
    k=1;
    for i=1:row1
        if(cpst_o(i,1)~=0)
            cpst(k,1)=cpst_o(i,1);
            cpst(k,2)=cpst_o(i,2);
            k=k+1;
        end
    end

    %determine state
    row1=length(cpst);
    for i=1:npart
        particle(i).st(tt-1)=0;
        for j=1:row1
            if(i==cpst(j,1))
                particle(i).st(tt-1)=1;
            elseif(i==cpst(j,2))
                particle(i).st(tt-1)=2;
            end
        end
    end


    [complexct(tt-1),~]=size(cpst);
    %now walk
    for i=1:npart
        for lch=1:area
              if ((lattice(lch).x==particle(i).xp(tt-1)) && (lattice(lch).y==particle(i).yp(tt-1)))
                  oc(1)=lattice(lch).occu;
                  ltid(1)=lch;
              elseif((lattice(lch).x==particle(i).xp(tt-1)) && (lattice(lch).y==particle(i).yp(tt-
```

```
1)+1))
                oc(2)=lattice(lch).occu;
                ltid(2)=lch;
            elseif((lattice(lch).x==particle(i).xp(tt-1)) && (lattice(lch).y==particle(i).yp(tt-1)-1))
                oc(3)=lattice(lch).occu;
                ltid(3)=lch;
            elseif((lattice(lch).x==particle(i).xp(tt-1)-1) && (lattice(lch).y==particle(i).yp(tt-1)))
                oc(4)=lattice(lch).occu;
                ltid(4)=lch;
            elseif((lattice(lch).x==particle(i).xp(tt-1)+1) && (lattice(lch).y==particle(i).yp(tt-
1)))
                oc(5)=lattice(lch).occu;
                ltid(5)=lch;
            elseif((lattice(lch).x==particle(i).xp(tt-1)) && (lattice(lch).y==1))
                oc(6)=lattice(lch).occu;
                ltid(6)=lch;
            elseif((lattice(lch).x==particle(i).xp(tt-1)) && (lattice(lch).y==b))
                oc(7)=lattice(lch).occu;
                ltid(7)=lch;
            elseif((lattice(lch).x==1) && (lattice(lch).y==particle(i).yp(tt-1)))
                oc(8)=lattice(lch).occu;
                ltid(8)=lch;
            elseif((lattice(lch).x==l) && (lattice(lch).y==particle(i).yp(tt-1)))
                oc(9)=lattice(lch).occu;
                ltid(9)=lch;
            end
        end
        %walk for gag and snap or halo non bonded
        if(particle(i).st(tt-1)==0)
        %if((particle(i).type==3) || (particle(i).type==1 && (particle(i).parttype(tt-1,1)~=2 ||
particle(i).parttype(tt-1,2)~=2)) ||(particle(i).type==1 && (particle(i).parttype(tt-1,1)==2 &&
particle(i).parttype(tt-1,2)==2) || (particle(i).type==1 && (particle(i).parttype(tt-1,1)==1 ||
particle(i).parttype(tt-1,2)==1))) || (particle(i).type==2 && (particle(i).parttype(tt-1,1)~=1 ||
particle(i).parttype(tt-1,2)~=1)) || (particle(i).type==2 && (particle(i).parttype(tt-1,1)==1 &&
particle(i).parttype(tt-1,2)==1)) || (particle(i).type==2 && (particle(i).parttype(tt-1,1)==2 ||
particle(i).parttype(tt-1,2)==2)))
            chx=mod(particle(i).xp(tt-1),2);
            chy=mod(particle(i).yp(tt-1),2);
            toss=rand;
            if(toss>=0 && toss<=(1/3))
                if(particle(i).yp(tt-1)+1<=b && oc(2)<3)
                    particle(i).xp(tt)=particle(i).xp(tt-1);
                    particle(i).yp(tt)=particle(i).yp(tt-1)+1;
                    particle(i).ts(tt)=tt;
                    lattice(ltid(2)).occu=lattice(ltid(2)).occu+1;
                    lattice(ltid(1)).occu=lattice(ltid(1)).occu-1;
                elseif(particle(i).yp(tt-1)+1>b && oc(6)<3)
                    particle(i).xp(tt)=particle(i).xp(tt-1);
                    particle(i).yp(tt)=1;
                    particle(i).ts(tt)=tt;
                    lattice(ltid(6)).occu=lattice(ltid(6)).occu+1;
                    lattice(ltid(1)).occu=lattice(ltid(1)).occu-1;
                elseif((particle(i).yp(tt-1)+1<=b && oc(2)==3) || (particle(i).yp(tt-1)+1>b &&
oc(6)==3))
                    particle(i).xp(tt)=particle(i).xp(tt-1);
                    particle(i).yp(tt)=particle(i).yp(tt-1);
```

```
            particle(i).ts(tt)=tt;
        end
    elseif (toss>(1/3) && toss<=(2/3))
        if(particle(i).yp(tt-1)-1>=1 && oc(3)<3)
            particle(i).xp(tt)=particle(i).xp(tt-1);
            particle(i).yp(tt)=particle(i).yp(tt-1)-1;
            particle(i).ts(tt)=tt;
            lattice(ltid(3)).occu=lattice(ltid(3)).occu+1;
            lattice(ltid(1)).occu=lattice(ltid(1)).occu-1;
        elseif(particle(i).yp(tt-1)-1<1 && oc(7)<3)
            particle(i).xp(tt)=particle(i).xp(tt-1);
            particle(i).yp(tt)=b;
            particle(i).ts(tt)=tt;
            lattice(ltid(7)).occu=lattice(ltid(7)).occu+1;
            lattice(ltid(1)).occu=lattice(ltid(1)).occu-1;
        elseif((particle(i).yp(tt-1)-1>=1 && oc(3)==3) || (particle(i).yp(tt-1)-1<1 &&
oc(7)==3))
            particle(i).xp(tt)=particle(i).xp(tt-1);
            particle(i).yp(tt)=particle(i).yp(tt-1);
            particle(i).ts(tt)=tt;
        end
    elseif (toss>(2/3) && toss<=1)
        if((chx==0 && chy==1) || (chx==1 && chy==0))
            if(particle(i).xp(tt-1)-1>=1 && oc(4)<3)
                particle(i).xp(tt)=particle(i).xp(tt-1)-1;
                particle(i).yp(tt)=particle(i).yp(tt-1);
                particle(i).ts(tt)=tt;
                lattice(ltid(4)).occu=lattice(ltid(4)).occu+1;
                lattice(ltid(1)).occu=lattice(ltid(1)).occu-1;
            elseif(particle(i).xp(tt-1)-1<1 && oc(9)<3)
                particle(i).xp(tt)=l;
                particle(i).yp(tt)=particle(i).yp(tt-1);
                particle(i).ts(tt)=tt;
                lattice(ltid(9)).occu=lattice(ltid(9)).occu+1;
                lattice(ltid(1)).occu=lattice(ltid(1)).occu-1;
            elseif((particle(i).xp(tt-1)-1>=1 && oc(4)==3) || (particle(i).xp(tt-1)-1<1 &&
oc(9)==3))
                particle(i).xp(tt)=particle(i).xp(tt-1);
                particle(i).yp(tt)=particle(i).yp(tt-1);
                particle(i).ts(tt)=tt;
            end
        elseif((chx==0 && chy==0) || (chx==1 && chy==1))
            if(particle(i).xp(tt-1)+1<=l && oc(5)<3)
                particle(i).xp(tt)=particle(i).xp(tt-1)+1;
                particle(i).yp(tt)=particle(i).yp(tt-1);
                particle(i).ts(tt)=tt;
                lattice(ltid(5)).occu=lattice(ltid(5)).occu+1;
                lattice(ltid(1)).occu=lattice(ltid(1)).occu-1;
            elseif(particle(i).xp(tt-1)+1>l && oc(8)<3)
                particle(i).xp(tt)=1;
                particle(i).yp(tt)=particle(i).yp(tt-1);
                particle(i).ts(tt)=tt;
                lattice(ltid(8)).occu=lattice(ltid(8)).occu+1;
                lattice(ltid(1)).occu=lattice(ltid(1)).occu-1;
            elseif((particle(i).xp(tt-1)+1<=l && oc(5)==3) || (particle(i).xp(tt-1)+1>l &&
oc(8)==3))
```

```
                particle(i).xp(tt)=particle(i).xp(tt-1);
                particle(i).yp(tt)=particle(i).yp(tt-1);
                particle(i).ts(tt)=tt;
            end
        end
    end

    %walk for snap or halo bonded
    elseif(particle(i).st(tt-1)==1)
    %elseif((particle(i).type==1 && (particle(i).parttype(tt-1,1)==2 || particle(i).parttype(tt-
1,2)==2)) || (particle(i).type==2 && (particle(i).parttype(tt-1,1)==1 || particle(i).parttype(tt-
1,2)==1)))
        sec=0;
        for j=1:complexct(tt-1)
            if(i==cpst(j,1))
                sec=cpst(j,2);
                %break;
            end
        end
        %check=1;
        chx=mod(particle(i).xp(tt-1),2);
        chy=mod(particle(i).yp(tt-1),2);
        toss=rand;
        if(toss>=0 && toss<=(1/3))
            if(particle(i).yp(tt-1)+1<=b && oc(2)<2)
                particle(i).xp(tt)=particle(i).xp(tt-1);
                particle(i).yp(tt)=particle(i).yp(tt-1)+1;
                particle(i).ts(tt)=tt;
                particle(sec).xp(tt)=particle(sec).xp(tt-1);
                particle(sec).yp(tt)=particle(sec).yp(tt-1)+1;
                particle(sec).ts(tt)=tt;
                lattice(ltid(2)).occu=lattice(ltid(2)).occu+2;
                lattice(ltid(1)).occu=lattice(ltid(1)).occu-2;
            elseif(particle(i).yp(tt-1)+1>b && oc(6)<2)
                particle(i).xp(tt)=particle(i).xp(tt-1);
                particle(i).yp(tt)=1;
                particle(i).ts(tt)=tt;
                particle(sec).xp(tt)=particle(sec).xp(tt-1);
                particle(sec).yp(tt)=1;
                particle(sec).ts(tt)=tt;
                lattice(ltid(6)).occu=lattice(ltid(6)).occu+2;
                lattice(ltid(1)).occu=lattice(ltid(1)).occu-2;
            elseif((particle(i).yp(tt-1)+1<=b && oc(2)>=2) || (particle(i).yp(tt-1)+1>b &&
oc(6)>=2))
                particle(i).xp(tt)=particle(i).xp(tt-1);
                particle(i).yp(tt)=particle(i).yp(tt-1);
                particle(i).ts(tt)=tt;
                particle(sec).xp(tt)=particle(sec).xp(tt-1);
                particle(sec).yp(tt)=particle(sec).yp(tt-1);
                particle(sec).ts(tt)=tt;
            end
        elseif (toss>(1/3) && toss<=(2/3))
            if(particle(i).yp(tt-1)-1>=1 && oc(3)<2)
                particle(i).xp(tt)=particle(i).xp(tt-1);
                particle(i).yp(tt)=particle(i).yp(tt-1)-1;
                particle(i).ts(tt)=tt;
```

```
            particle(sec).xp(tt)=particle(sec).xp(tt-1);
            particle(sec).yp(tt)=particle(sec).yp(tt-1)-1;
            particle(sec).ts(tt)=tt;
            lattice(ltid(3)).occu=lattice(ltid(3)).occu+2;
            lattice(ltid(1)).occu=lattice(ltid(1)).occu-2;
          elseif(particle(i).yp(tt-1)-1<1 && oc(7)<2)
            particle(i).xp(tt)=particle(i).xp(tt-1);
            particle(i).yp(tt)=b;
            particle(i).ts(tt)=tt;
            particle(sec).xp(tt)=particle(sec).xp(tt-1);
            particle(sec).yp(tt)=b;
            particle(sec).ts(tt)=tt;
            lattice(ltid(7)).occu=lattice(ltid(7)).occu+2;
            lattice(ltid(1)).occu=lattice(ltid(1)).occu-2;
          elseif((particle(i).yp(tt-1)-1>=1 && oc(3)>=2) || (particle(i).yp(tt-1)-1<1 &&
oc(7)>=2))
            particle(i).xp(tt)=particle(i).xp(tt-1);
            particle(i).yp(tt)=particle(i).yp(tt-1);
            particle(i).ts(tt)=tt;
            particle(sec).xp(tt)=particle(sec).xp(tt-1);
            particle(sec).yp(tt)=particle(sec).yp(tt-1);
            particle(sec).ts(tt)=tt;
          end
        elseif (toss>(2/3) && toss<=1)
          if((chx==0 && chy==1) || (chx==1 && chy==0))
            if(particle(i).xp(tt-1)-1>=1 && oc(4)<2)
              particle(i).xp(tt)=particle(i).xp(tt-1)-1;
              particle(i).yp(tt)=particle(i).yp(tt-1);
              particle(i).ts(tt)=tt;
              particle(sec).xp(tt)=particle(sec).xp(tt-1)-1;
              particle(sec).yp(tt)=particle(sec).yp(tt-1);
              particle(sec).ts(tt)=tt;
              lattice(ltid(4)).occu=lattice(ltid(4)).occu+2;
              lattice(ltid(1)).occu=lattice(ltid(1)).occu-2;
            elseif(particle(i).xp(tt-1)-1<1 && oc(9)<2)
              particle(i).xp(tt)=l;
              particle(i).yp(tt)=particle(i).yp(tt-1);
              particle(i).ts(tt)=tt;
              particle(sec).xp(tt)=l;
              particle(sec).yp(tt)=particle(sec).yp(tt-1);
              particle(sec).ts(tt)=tt;
              lattice(ltid(9)).occu=lattice(ltid(9)).occu+2;
              lattice(ltid(1)).occu=lattice(ltid(1)).occu-2;
            elseif((particle(i).xp(tt-1)-1>=1 && oc(4)>=2) || (particle(i).xp(tt-1)-1<1 &&
oc(9)>=2))
              particle(i).xp(tt)=particle(i).xp(tt-1);
              particle(i).yp(tt)=particle(i).yp(tt-1);
              particle(i).ts(tt)=tt;
              particle(sec).xp(tt)=particle(sec).xp(tt-1);
              particle(sec).yp(tt)=particle(sec).yp(tt-1);
              particle(sec).ts(tt)=tt;
            end
          elseif((chx==0 && chy==0) || (chx==1 && chy==1))
            if(particle(i).xp(tt-1)+1<=l && oc(5)<2)
              particle(i).xp(tt)=particle(i).xp(tt-1)+1;
              particle(i).yp(tt)=particle(i).yp(tt-1);
```

```
                particle(i).ts(tt)=tt;
                particle(sec).xp(tt)=particle(sec).xp(tt-1)+1;
                particle(sec).yp(tt)=particle(sec).yp(tt-1);
                particle(sec).ts(tt)=tt;
                lattice(ltid(5)).occu=lattice(ltid(5)).occu+2;
                lattice(ltid(1)).occu=lattice(ltid(1)).occu-2;
            elseif(particle(i).xp(tt-1)+1>l && oc(8)<2)
                particle(i).xp(tt)=1;
                particle(i).yp(tt)=particle(i).yp(tt-1);
                particle(i).ts(tt)=tt;
                particle(sec).xp(tt)=1;
                particle(sec).yp(tt)=particle(sec).yp(tt-1);
                particle(sec).ts(tt)=tt;
                lattice(ltid(8)).occu=lattice(ltid(8)).occu+2;
                lattice(ltid(1)).occu=lattice(ltid(1)).occu-2;
            elseif((particle(i).xp(tt-1)+1<=l && oc(5)>=2) || (particle(i).xp(tt-1)+1>l &&
oc(8)>=2))
                particle(i).xp(tt)=particle(i).xp(tt-1);
                particle(i).yp(tt)=particle(i).yp(tt-1);
                particle(i).ts(tt)=tt;
                particle(sec).xp(tt)=particle(sec).xp(tt-1);
                particle(sec).yp(tt)=particle(sec).yp(tt-1);
                particle(sec).ts(tt)=tt;
            end
          end
        end

      end

    end
    %determine the partners and partner idx
    cp=0;
    for i=1:npart
      cp=0;
      for j=1:npart
        if(particle(i).xp(tt)==particle(j).xp(tt) && particle(i).yp(tt)==particle(j).yp(tt) && j~=i)
          cp=cp+1;
          particle(i).parttype(tt,cp)=particle(j).type;
          particle(i).partidx(tt,cp)=j;
        end
      end
      if(cp==1)
        particle(i).parttype(tt,2)=0;
        particle(i).partidx(tt,2)=0;
      elseif(cp==0)
        particle(i).parttype(tt,1)=0;
        particle(i).partidx(tt,1)=0;
        particle(i).parttype(tt,2)=0;
        particle(i).partidx(tt,2)=0;
      end
    end
%     %update lattice occupancy
%     for i=1:area
%       lattice(i).occu=0;
%       for j=1:npart
%         if(lattice(i).x==particle(j).xp(1) && lattice(i).y==particle(j).yp(1))
```

```
%                 lattice(i).occu=lattice(i).occu+1;
%             end
%         end
%     end
end

%plot lattice points
figure(1);
for i=1:area
    plot(lattice(i).x,lattice(i).y,'.b');
    hold on;
end
%plot initial position
for i=1:npart
    plot(particle(i).xp(1),particle(i).yp(1),'*r');
end
hold off

%plot complex numbers vs time
figure(2);
plot(particle(1).ts(1:tt-1),complexct);
```

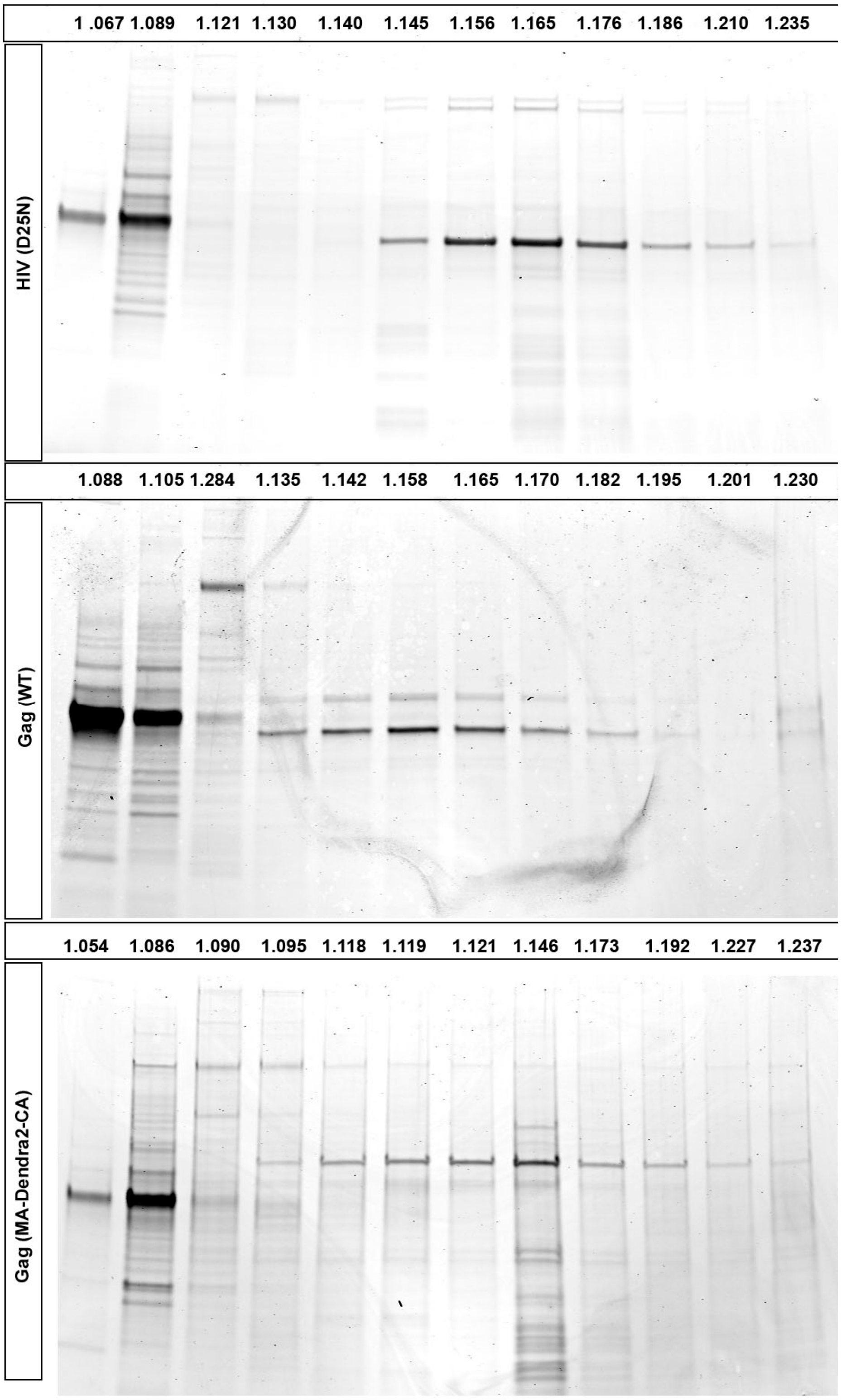

Fig. S1. Fractions from 20-60% sucrose gradient of HIV(D25N), Gag(MA-Dendra2-CA) and Gag(WT) VLPs analyzed by protein stain. Density of each fraction is typed on top of each lane.

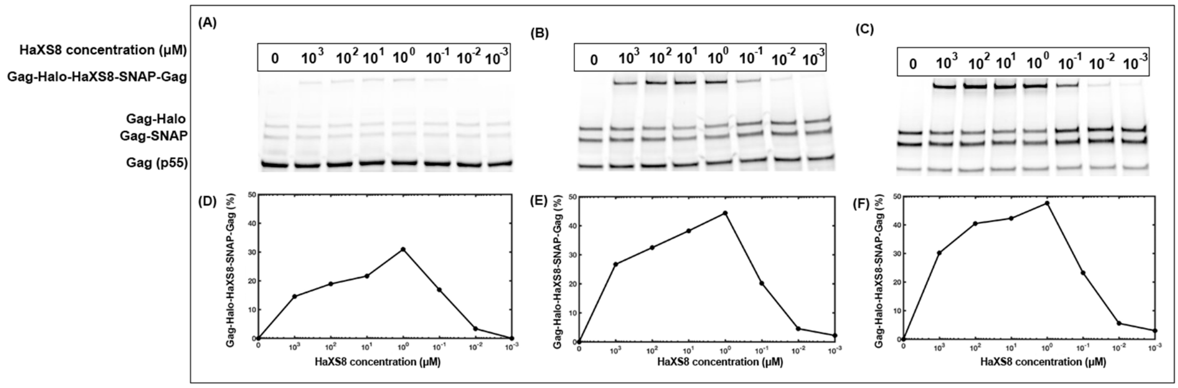

Fig. S2. The VLPs were treated with HAXS8 of the indicated concentration and incubated in 33C. (A) *shows western blot analysis of VLPs incorporating 80% Gag, 10% Gag-SNAP and 10% Gag-Halo treated with HAXS8 of indicated concentration. (B) shows western blot analysis of VLPs incorporating 60% Gag, 20% Gag-SNAP and 20% Gag-Halo treated with HAXS8 of indicated concentration. (C)shows western blot analysis of VLPs incorporating 20% Gag, 40% Gag-SNAP and 40% Gag-Halo treated with HAXS8 of indicated concentration. (D) indicates the density of SNAP-Halo complex obtained in each case of (A). (E) indicates the density of SNAP-Halo complex obtained in each case of (B). (F) indicates the density of SNAP-Halo complex obtained in each case of (C).*
